# Mapping and mutation of the conserved DNA polymerase interaction motif (DPIM) located in the C-terminal domain of fission yeast DNA polymerase δ subunit Cdc27

**DOI:** 10.1186/1471-2199-5-21

**Published:** 2004-12-03

**Authors:** Fiona C Gray, J Richard G Pohler, Emma Warbrick, Stuart A MacNeill

**Affiliations:** 1 Wellcome Trust Centre for Cell Biology, University of Edinburgh, Michael Swann Building, King's Buildings, Mayfield Road, Edinburgh EH9 3JR, UK; 2 Department of Surgery and Molecular Oncology, University of Dundee, Ninewells Hospital and Medical School, Dundee DD1 9SY, UK

## Abstract

**Background:**

DNA polymerases α and δ play essential roles in the replication of chromosomal DNA in eukaryotic cells. DNA polymerase α (Pol α)-primase is required to prime synthesis of the leading strand and each Okazaki fragment on the lagging strand, whereas DNA polymerase δ (Pol δ) is required for the elongation stages of replication, a function it appears capable of performing on both leading and lagging strands, at least in the absence of DNA polymerase ε (Pol ε).

**Results:**

Here it is shown that the catalytic subunit of Pol α, Pol1, interacts with Cdc27, one of three non-catalytic subunits of fission yeast Pol δ, both *in vivo *and *in vitro*. Pol1 interacts with the C-terminal domain of Cdc27, at a site distinct from the previously identified binding sites for Cdc1 and PCNA. Comparative protein sequence analysis identifies a protein sequence motif, called the DNA polymerase interaction motif (DPIM), in Cdc27 orthologues from a wide variety of eukaryotic species, including mammals. Mutational analysis shows that the DPIM in fission yeast Cdc27 is not required for effective DNA replication, repair or checkpoint function.

**Conclusions:**

The absence of any detectable phenotypic consequences arising from mutation of the DPIM suggests that despite its evolutionary conservation, the interaction between the two polymerases mediated by this motif is a non-essential one.

## Background

Three conserved DNA polymerase enzymes whose activities are essential for complete chromosomal DNA replication have been identified through biochemical studies in mammalian systems [1] and combined genetic and biochemical studies in yeast [2]. During S-phase, the DNA polymerase α-primase complex synthesises the short RNA-DNA segment that is used to prime synthesis of the leading strand at the chromosomal replication origin and synthesis of each Okazaki fragment on the lagging strand. The short RNA segment is synthesised by the primase and then extended by 10–20 nucleotides by Pol α. The 3' end of the RNA-DNA primer is recognised by replication factor C (RFC), which displaces the Pol α-primase complex and catalyses the loading of the sliding clamp PCNA at the primer-template junction. PCNA then acts as a processivity factor for the Pol δ and/or Pol ε enzymes. The exact roles played by Pol δ and Pol ε remain unclear (for a recent perspective, see ref. [3] and references therein) but Pol δ is most likely responsible for lagging strand replication and may also play a role on the leading strand. Yeast lacking Pol ε catalytic activity are viable but are slow growing and somewhat impaired in chromosome replication [4-7]. In such cells, Pol δ is thought to perform the bulk of nascent DNA chain elongation, raising the possibility that this enzyme performs a similar function in wild-type cells. If this is the case, Pol ε could have a specialised role, at replication origins for example, or in the replication of sites of sister chromosome cohesion [3].

Each of the three essential polymerases is a multi-subunit entity, comprising a large catalytic subunit and a number of smaller subunits that are presumed to play either regulatory or structural roles [2]. Little is known of the biochemical functions of the smaller subunits but in yeast most are, like the three catalytic subunits, essential proteins. In the fission yeast *Schizosaccharomyces pombe*, the Pol α-primase and Pol δ complexes are both heterotetrameric in structure [2]. The catalytic subunits of the two complexes, Pol1 and Pol3 respectively, are members of the B family of DNA polymerases typified by bacteriophage T4 polymerase or Pfu [8]. Both complexes also contain related B-subunits. These proteins are members of a larger family of phosphotransferase and nuclease enzymes [9,10], although no enzymatic activity has been detected for either of the DNA polymerase subunits. In fission yeast, the B-subunit of Pol δ is the Cdc1 protein. Fission yeast Pol δ also contains two further subunits: the C-subunit Cdc27, which functions in part to link the polymerase to PCNA [11,12], and the D-subunit Cdm1. Of the four Pol δ subunits, only Cdm1 is non-essential [13]. Orthologues of all four of these proteins (Pol3, Cdc1, Cdc27 and Cdm1) make up mammalian Pol δ [14,15].

Perhaps surprisingly, there have been few reports of physical interactions between the various polymerase enzymes believed to be present at the eukaryotic replication fork. One exception to this comes from large-scale functional analysis of the budding yeast proteome where an interaction was uncovered between the catalytic subunit of the Pol α-primase complex, Pol1, and the C-subunit of the Pol δ complex in budding yeast, Pol32 [16]. Interaction between these proteins was detected using the two-hybrid system, raising the possibility that the interaction was not a direct one, but was instead mediated via a third protein factor or complex. Recently, the two-hybrid result was independently confirmed and a direct interaction between the budding yeast Pol α-primase and Pol δ demonstrated by mixing of the purified enzyme complexes followed by co-immunoprecipitation [17]. By this method, Pol α-primase and Pol δ were shown interact in a Pol32-dependent manner [17]. A form of Pol δ containing a mutant Pol32 protein lacking a 40 amino acid region of the C-terminal domain (Pol32-8, lacking amino acids 270–309) was also shown to be unable to co-immunoprecipitate with Pol α-primase [17], providing the first indication of the location of the Pol α binding site on Pol32.

In this paper, it is shown that the orthologues of the budding yeast Pol1 and Pol32 proteins in fission yeast, Pol1 and Cdc27 respectively, also interact in the two-hybrid system. It is also shown that these proteins are capable of interacting directly with one another, *in vitro*, using purified recombinant proteins and peptides. Interaction is also seen between the human Cdc27 and Pol1 homologues, p66 and p180. The binding site for Pol1 maps to the extended C-terminal domain of the Cdc27 protein and requires the presence of a short protein sequence motif, which we have designated the DPIM, for DNA polymerase interaction motif. This short sequence is conserved in Cdc27 orthologues from a wide variety of eukaryotic species. Despite this evolutionary conservation, mutational inactivation of the Pol1 binding motif does not affect Cdc27 function *in vivo*. The implications of these results are discussed.

## Results

### Interaction between fission yeast Pol1 and Cdc27

**Figure 1:**
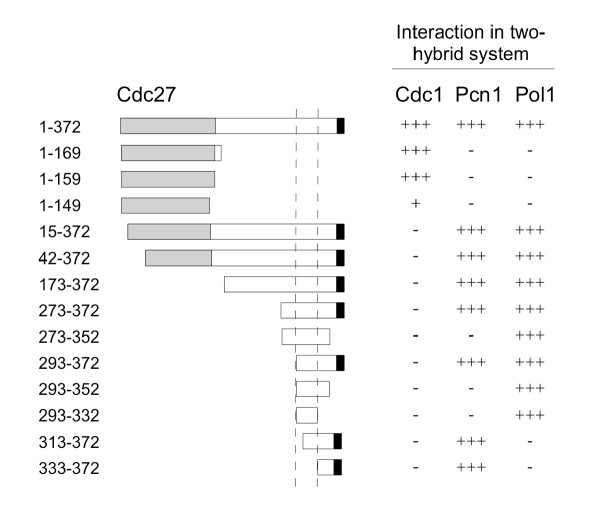
**Mapping of the Pol1 interaction site on Cdc27 using the two-hybrid system. **Full-length and thirteen truncated Cdc27 proteins, expressed as Gal4 transcription activation domain fusions, were tested for their ability to interact with Cdc1-LexA, Pcn1-LexA and Pol1(278–512)-LexA baits in *S. cerevisiae *CTY10-5d. Interactions were monitored by β-galactosidase activity, as described in Material and methods, and are indicated as +++ (strong interaction, typically 80–100 Miller units of β-galactosidase activity), + (weak interaction, <20 Miller units), – (no interaction detectable above background, <1 Miller unit). The area between the broken vertical lines indicates the minimal Pol1 binding region, corresponding to amino acids 293–332 of Cdc27. The grey box indicates the minimal Cdc1 binding domain, amino acids 1–160; the black box represents the Pcn1 (PCNA) binding motif (amino acids 362–372).

To test for interaction between fission yeast Pol1 and Cdc27, the two-hybrid system was used. Full-length Cdc27 fused to the activation domain (AD) of the yeast Gal4 protein was tested for its ability to interact with amino acids 278–527 of fission yeast Pol1 fused to the DNA binding domain of the bacterial transcription factor LexA (see Materials and methods for details). Amino acids 278–527 correspond to the smallest region of budding yeast Pol1 shown to interact with Pol32 [16]. Reporter gene induction, measured by β-galactosidase activity assay, was observed in the presence of the Gal4 AD-Cdc27 and LexA-Pol1-278-527 (LexA-Pol1) proteins, but not when either protein alone was present (Figure 1 and Table 1A, upper part). Thus, as in budding yeast, the catalytic subunit of Pol α is able to interact with the C-subunit of Pol δ.

**Table 1:** Two-hybrid analysis. Prey and bait proteins were expressed as Gal4 AD and LexA BD fusions from pACT2 and pBTM116 respectively. Positive interactions corresponded to 50–100 Miller units (++) or 10–20 Miller units (+) of β-galactosidase activity. Negative interactions corresponded to < 2 units. See text for details.

Prey	Bait	Bridge	Interaction
A. Fission yeast proteins			
Cdc27	-	-	-
-	Pol1(278–527)	-	-
Cdc27	Pol1(278–527)	-	++
Pcn1	Pol1(278–527)	-	-
Pcn1	-	Cdc27	-
-	Pol1(278–527)	Cdc27	-
Pcn1	Pol1(278–527)	Cdc27	+
B. Human proteins			
-	Pol α (291–540)	-	-
p66 (253–466)	-	-	-
p66 (356–466)	-	-	-
p66 (253–466)	Pol α (291–540)	-	++
p66 (356–466)	Pol α (291–540)	-	++
C. Cross-species interactions			
-	Pol1(278–527)	-	-
p66 (253–466)	Pol1(278–527)	-	++
p66 (356–466)	Pol1(278–527)	-	++
Cdc27	Pol α (291–540)	-	-
D. Truncated Pol1 proteins			
Cdc27	Pol1(278–527)	-	++
Cdc27	Pol1(328–527)	-	-
Cdc27	Pol1(278–477)	-	-
Cdc27	Pol1(278–487)	-	-
Cdc27	Pol1(278–497)	-	-
Cdc27	Pol1(278–507)	-	-
Cdc27	Pol1(328–477)	-	-
Cdc27	Pol1-TS13(278–527)	-	-

### Mapping the Pol1 binding site on Cdc27

Previously, minimal Cdc1 and PCNA binding regions on Cdc27 have been mapped [11,12,18]. Cdc1 binds within the globular 160 amino acid N-terminal domain of the 372 amino acid Cdc27 protein, whereas PCNA binds at the extreme C-terminus of Cdc27 at a conserved p21^Cip1^-like PCNA binding sequence, the PIP box. To map the Pol1 binding site, a series of thirteen truncated Cdc27 proteins fused to the Gal4 AD were tested against LexA-Pol1 in the two-hybrid system. The results of this analysis are shown in Figure 1. Pol1 binds within the extended C-terminal domain of Cdc27, with the smallest construct capable of binding spanning a forty amino acid region, from amino acids 293–332. Therefore, the Pol1 binding region is distinct from both the globular N-terminal domain and C-terminal PIP box. In support of this, two-hybrid analysis showed that Cdc27 could bind to both Pol1 and Pcn1 (PCNA) proteins simultaneously: Gal4 AD-Pcn1 and LexA-Pol1 fusion proteins (which do not interact in the two-hybrid system) could be brought together by co-expression of Cdc27 (Table 1A, lower part).

### Cdc27-Pol1 interactions with recombinant proteins

**Figure 2:**
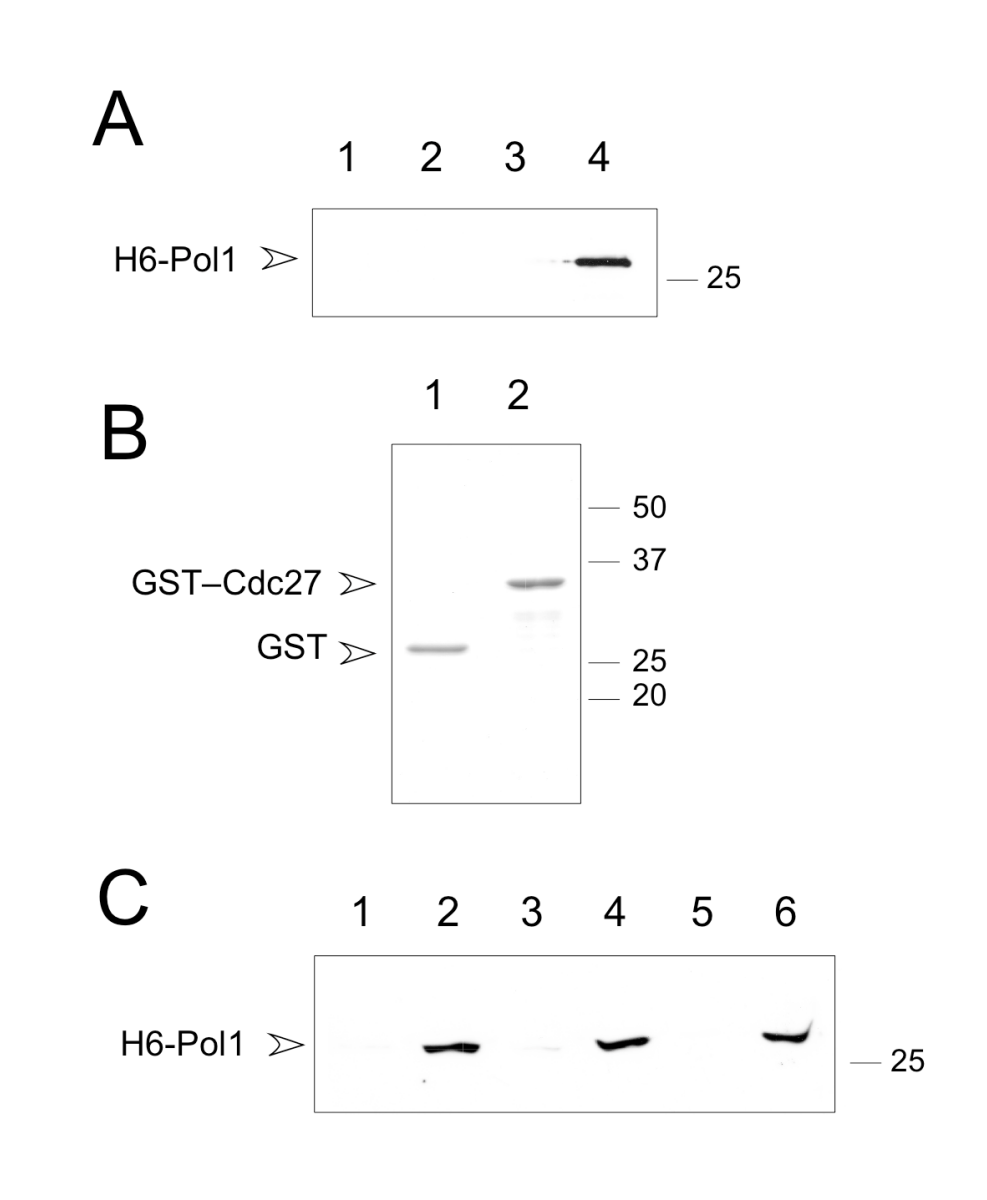
**Direct interaction between recombinant Pol1 (278–512) and Cdc27 proteins. A. **Expression of H6-Pol1 (278–512) in *E. coli *detected by immunoblotting using anti-MRGS antibodies. Total protein extracts prepared from cells carrying the control plasmid pQE32-Pol1 (278–512)-REV (lanes 1, 2), in which the relevant *pol1*^+ ^sequence is present in the wrong orientation, or pQE32-Pol1 (278–512) (lanes 3, 4), either before (lanes 1, 3) or after (lanes 2, 4) induction of protein expression by addition of IPTG. The position of the 25 kDa marker is shown. **B. **Purified GST and GST – Cdc27 (273–352) proteins detected by Coomassie staining following SDS-PAGE. **C. **Binding assays using GST – Cdc27 fusion proteins. Bound H6-Pol1 was detected by Western blotting as in part A. Lanes 1, 3, 5: binding of H6-Pol1 to GST. Lanes 2, 4, 6: binding of binding of H6-Pol1 to GST – Cdc27 (273–352). In each case the bound fraction corresponds to approximately 15–20% of the input. Lanes 1 and 2: assay performed in PBS containing 0.1% Triton X100; lanes 3 and 4: PBS containing 0.25% Triton X100; lanes 5 and 6: PBS containing 0.5% Triton X100. In the absence of detergent Pol1 binds equally well to both GST and GST-Cdc27 (273–352) proteins.

In order to test whether the interaction between Pol1 and Cdc27 was a direct one, purified recombinant Cdc27 and Pol1 proteins were assayed for their ability to interact *in vitro*. Purified GST-Cdc27-273-352 fusion protein [11] was tested for its ability to pull-down purified recombinant hexahistidine-tagged Pol1 (amino acids 278–527). The results (Figure 2) mirror those seen with the two-hybrid assays and provide the first evidence that the interaction between the Cdc27 and Pol1 proteins is a direct one, rather than being mediated via a third protein or set of proteins, such as one of the other subunits of the Pol α-primase or Pol δ complexes.

### Pol1 binding site sequence

**Figure 3:**
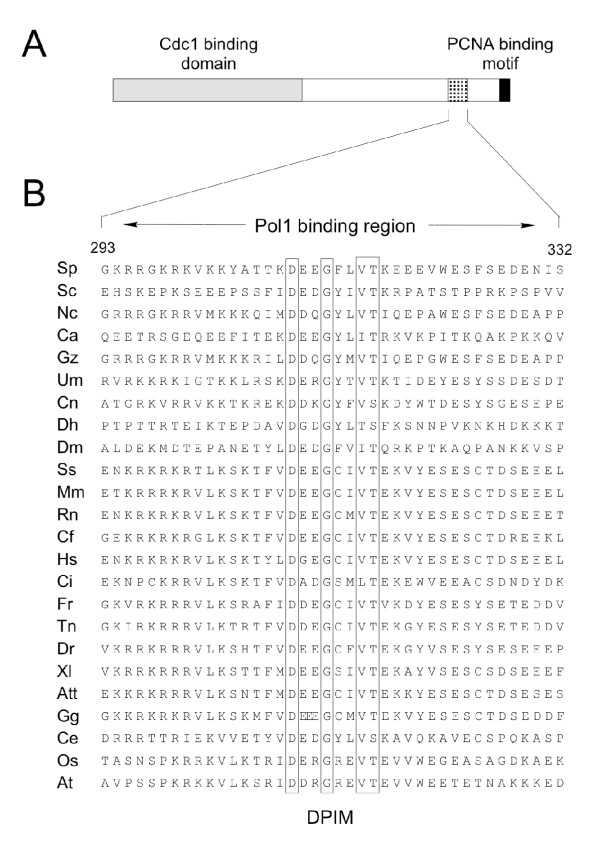
**Sequence alignment of Pol1 interacting region from fission yeast Cdc27 and homologues in other eukaryotic species. **Conserved residues are boxed. Abbreviations and NCBI sequence accession numbers: Sp (*S. pombe*, P30261), Sc (*S. cerevisiae*, P47110), Nc (*Neurospora crassa*, XP_328704), Ca (*Candida albicans*, EAK99562), Gz (*Gibberella zeae*, XP_384433), Um (*Ustilago maydis*, XP_401381), Cn (*Cryptococcus neoformans*, EAL21672), Dh (*Debaryomyces hansenii*, CAG87841), Dm (*Drosophila melanogaster*, AAD38629), Ss (*Sus scrofa*, BF078337), Mm (*Mus musculus*, Q9EQ28), Rn (*Rattus norvegicus*, XP_215011), Cf (*Canis familiaris*, CF411342), Hs (*Homo sapiens*, Q15054), Ci (*Ciona intestinalis*, AK114729), Fr (*Fugu rubripes*, protein sequence derived by translation of clone M001240 at ), Tn (*Tetraodon nigroviridis*, CAF97746), Dr (*Danio rerio*, AAH76031), Xl (*Xenopus laevis*, BAC82197), Att (*Ambystoma tigrinum tigrinum*, CN059104), Gg (*Gallus gallus*, BU121824 – note the additional glutamate residue within the DPIM), Ce (*C. elegans*, Q21730), Os (*Oryza sativa*, NP_913217), and At (*Arabadopsis thaliana*, C96815). Note that in only a few cases (Sp, Sc, Hs, Mm, Xl) have the identities of these putative Cdc27 proteins been confirmed via purification and characterisation of Pol δ. However, all the sequences shown possess a canonical PCNA binding motif at or near their C-terminal ends (Q -- I -- FF), in common with the *bone fide *Cdc27 proteins.

Previously, Cdc27 homologues from fission yeast [11,18], budding yeast [19,20], human, mouse [21], and *Xenopus *[15] have been identified and characterised. To extend this family, BLAST [22] and ψ-BLAST searches [23,24] were performed to identify putative Cdc27 family members from other eukaryotic species. In all, 24 protein sequences were identified, from organisms as diverse as vertebrates, worms, fungi and plants. Protein sequence alignments (Figure 3) of the C-terminal regions of these proteins identified a set of highly conserved amino acids that form a putative DNA polymerase interaction motif (or DPIM) with the consensus sequence D(D/E)-G -- (V/I)(T/S). The sequences flanking the DPIM are generally highly charged in character and some sequence conservation (particularly of charged amino acids) is apparent in these regions. Mutagenesis and peptide binding studies (described below) suggest that, in addition to the DPIM, some of these sequences may also play a role in binding Pol1 (see Discussion).

### Interaction between human Pol1 and Cdc27 orthologues

To examine whether the Pol1-Cdc27 (Pol1-Pol32) interaction observed in the yeasts was also conserved in higher eukaryotes, the catalytic subunit of human Pol α and the human Cdc27 orthologue p66/KIA00039 were assayed for interaction using the two-hybrid system. Amino acids 291–540 of the human Pol α catalytic subunit, corresponding to the minimum Cdc27 binding region (amino acids 278–527) in fission yeast Pol1, were expressed as a LexA fusion alongside Gal4 activation domain fusions of either the entire C-terminal domain of human p66 (amino acids 253–466) or the C-terminal 111 amino acids only (356–466). Interactions were tested by β-galactosidase assay. Both p66 constructs bound to Pol α (Table 1B), indicating that the DNA polymerase interaction is a conserved feature. In addition, it was observed that human p66 was able to interact with fission yeast Pol1 (Table 1C), suggesting that conserved sequences (or structural features) within the Cdc27/p66 C-terminal region, such as the DPIM, were important for the interaction.

### Mutational analysis of Pol1 binding site on Cdc27

**Figure 4:**
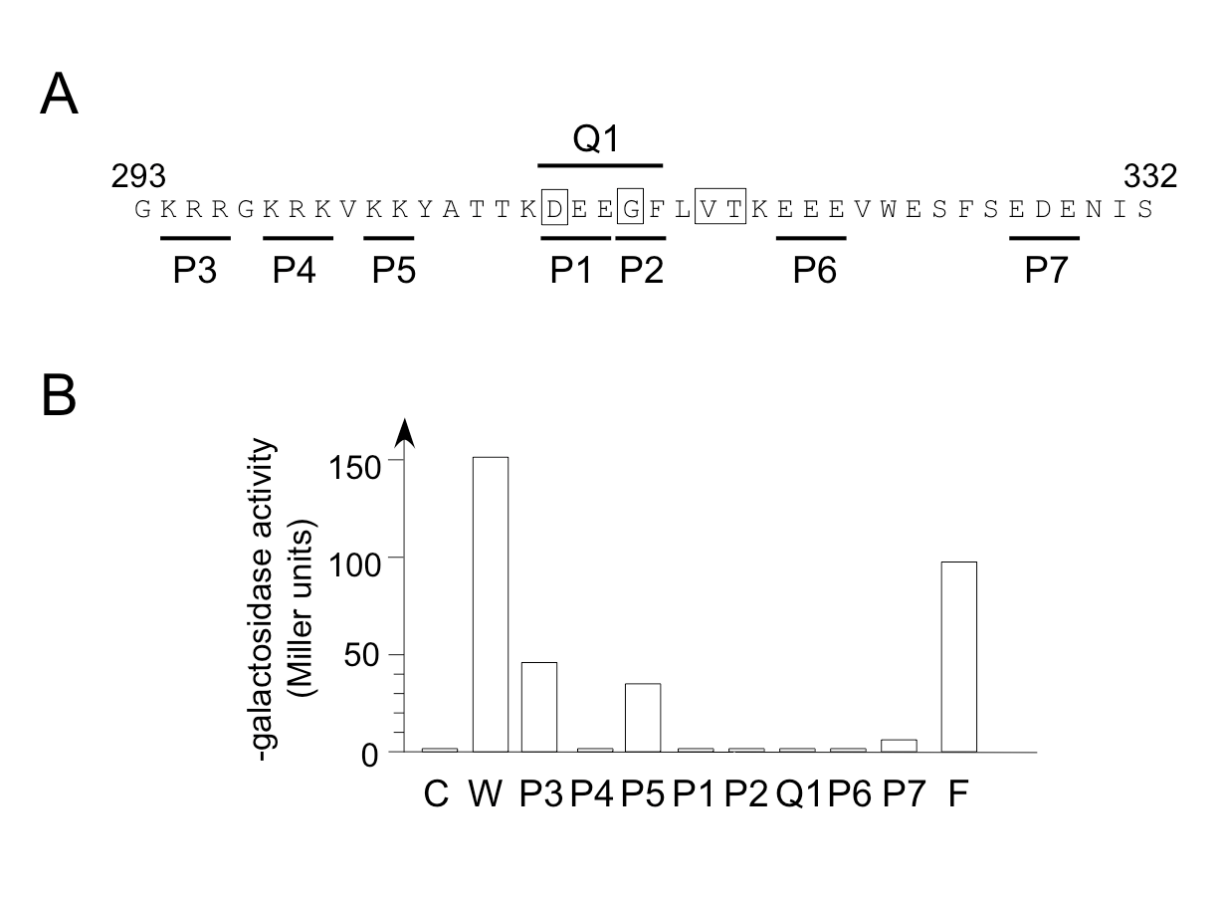
**Mutational analysis of Pol1 binding domain on Cdc27. A. **Summary schematic of minimal Pol1 binding region on Cdc27, showing conserved amino acids (boxed) and the eight mutant alleles P1 through P7 and Q1. Each mutant sees two or three adjacent residues being replaced with the same number of alanines. In the case of P1 and P3 through P7, adjacent basic (P3, P4 and P5) or acidic (P1, P6 and P7) amino acids are mutated. **B. **Quantitation of β-galactosidase activity in liquid cultures. Key: C (pACT2 vector); W (pACT-Cdc27-273-352), mutants P1 – P7, plus Q1 (mutated forms of pACT-Cdc27-273-352); F (pACT-Cdc27, i.e. full-length Cdc27 fused to Gal4).

Next, the importance for Pol1 binding of the conserved amino acids in the DPIM was examined. Eight mutant Cdc27 proteins (Cdc27-P1 through Cdc27-P7, and Cdc27-Q1, see Figure 4) were expressed as Gal4-Cdc27-273-352 fusion proteins and tested for their ability to bind to LexA-Pol1 (278–527) in the two-hybrid system. In each mutant protein one or more conserved amino acids is replaced with alanine. For example, the Cdc27-P1 mutant sees the conserved triplet DEE (residues 309–311) substituted with AAA. The results of this analysis are shown in Figure 4. Mutating the conserved amino acids of the DPIM (mutants Cdc27-P1, P2 and Q1) completely abolished Pol1 binding *in vivo *by Cdc27. Similar reductions were seen with the Cdc27-P4 and Cdc27-P6 mutants, where the mutated residues are located N-terminal and C-terminal to the DPIM respectively. The conserved amino acids of the DPIM are therefore essential for Pol1 binding by Cdc27, although sequences flanking the conserved motif also play a role. Only three of the eight mutant proteins (Cdc27-P3, P5 and P7) were able to interact with LexA-Pol1 (278–527), though the strength of the interaction was reduced to 30–40% of the wild-type value for Cdc27-P3 and Cdc27-P5 (both of which affect residues N-terminal to the conserved motif), and to ~ 10% of wild-type for Cdc27-P7 (located C-terminal to the conserved motif). Immunoblotting showed that all the mutant proteins were present in yeast protein extracts at the same level as the wild-type Gal4-Cdc27 fusion protein (data not shown).

**Figure 5:**
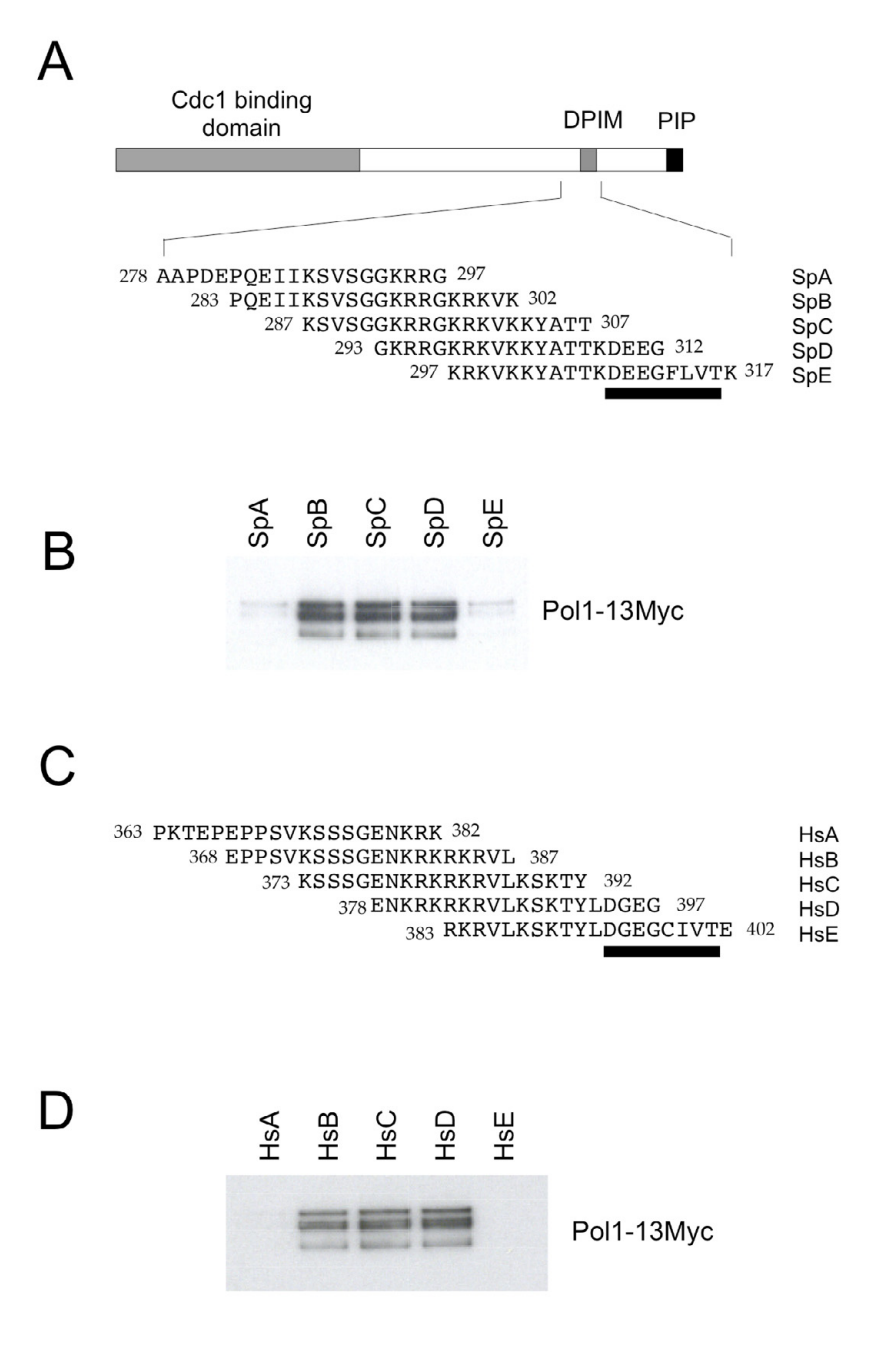
**Peptide binding studies. A. **Schematic of the structure of the Cdc27 protein (upper part) with the sequences of overlapping peptides SpA-SpE shown beneath. **B. **Immunoblot using anti-myc mAb showing pull-down of Pol1-13Myc protein from fission yeast protein extracts by Sp series peptides. **C. **Sequences of human peptide set HsA-HsE corresponding exactly to SpA-SpE above. **D. **Immunoblot using anti-myc mAb showing pull-down of Pol1-13Myc protein from fission yeast protein extracts by Hs series peptides.

The observation that sequences flanking the DPIM play a role in Pol1 binding was supported by the results of studies performed with a nested set of overlapping 20 mer peptides derived from the *S. pombe *Cdc27 sequence (see Figure 5A). The peptides were tested for their ability to bind to an epitope-tagged form of the Pol1 protein (Pol1-13Myc, see Materials and methods) in fission yeast protein extracts. As can be seen in Figure 5B, Pol1-13myc is precipitated by peptides SpB, SpC and SpD, but not SpA and SpE. Peptide SpC spans the groups of basic amino acids N-terminal to the conserved motif, including those shown to be required for Pol1 binding as defined by the Cdc27-P3 and -P5 mutants above, but does not include the conserved sequence DEEGFLVT indicating that, in this *in vitro *situation, the conserved amino acids are not absolutely required for Pol1 binding, in contrast to what is observed *in vivo*. Similar results were observed with peptides derived for the human p66 protein sequence (Figure 5C,5D), again illustrating the potential for cross-species interaction between human p66 and fission yeast Pol1 (see Table 1C).

### Mapping the Cdc27 binding site on Pol1

In an effort to map more precisely where within the Pol1 protein Cdc27 binds, several truncated forms of Pol1 (278–527) were constructed and tested as LexA fusions in the two-hybrid system (see Materials and methods for details). Removal of fifty amino acids from the N-terminus, or twenty amino acids from the C-terminus, of the Pol1 (278–527) protein was found to abolish the interaction with Cdc27 altogether (Table 1D).

Previously, the isolation of a temperature-sensitive mutant *pol1 *allele, *pol1-ts13*, had been reported [25]. Sequence analysis revealed that this allele differed from the wild-type *pol1*^+ ^by deletion of 9 bp from the ORF, resulting in loss of three amino acids (L470, S471, R472) from within the minimal Cdc27 binding domain defined above. In this study, the ability of a Pol1-TS13(278–527) bait to bind to Cdc27 was tested using the two-hybrid system. No interaction could be detected at a range of growth temperatures (Table 1D, and data not shown), indicating that amino acids 470–472 are required for Cdc27 binding by Pol1(278–527). To test whether overproduction of Cdc27 might rescue the temperature-sensitive phenotype of *pol1-ts13*, these cells were transformed with plasmid pREP3X-Cdc27 [18] and transformants plated at restrictive and semi-restrictive temperatures on thiamine-free medium, to induce high-level expression of *cdc27*^+ ^from the thiamine-repressible nmt promoter. No suppression of the *pol1-ts13 *phenotype was observed, however (data not shown). Indeed, no suppression was observed of any of three *pol1 *alleles that were analysed in this way, the others being *pol1-1 *[26] and *pol1-H4 *[27].

### Expression of DPIM mutants *in vivo*

To assay the *in vivo *role of the DPIM in Cdc27, four of the eight DPIM mutant alleles (*cdc27-P1 *through *cdc27-P4*) were cloned into plasmid pREP3X, 3' to the repressible nmt1 promoter [28,29], and transformed into a *cdc27*^+^/*cdc27::his7*^+ ^diploid strain. Transformant colonies were then induced to sporulate and the spores plated on media containing thiamine, to repress the nmt1 promoter. Under these conditions, residual low level expression from the nmt1 promoter ensures that the level of Cdc27 protein present in the cell is comparable to that seen in wild-type cells [12]. Analysis of the meiotic products showed that the mutant Cdc27 proteins were able to support growth of *cdc27Δ *haploid cells; indeed, no phenotypic defects were apparent (data not shown).

**Figure 6:**
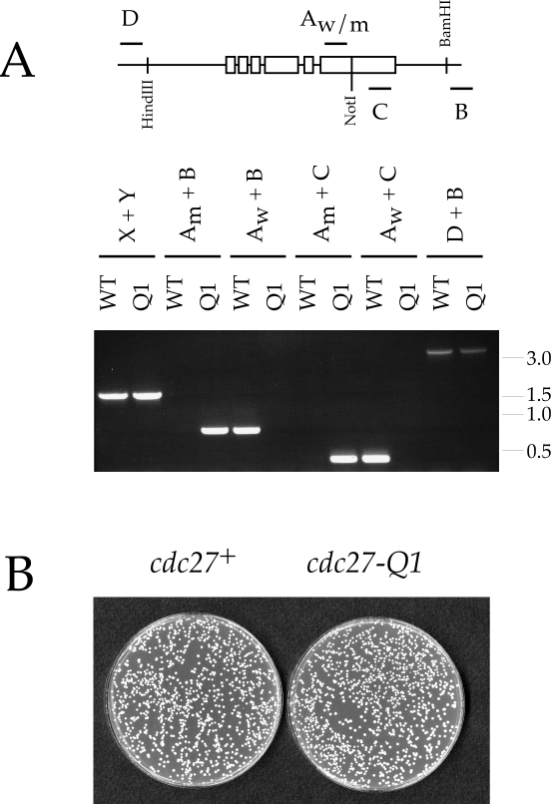
**Construction and analysis of *cdc27-Q1 *mutant yeast. A. **Upper part: Schematic of the *cdc27*^+ ^gene region (3.1 kb HindIII-BamHI region) showing location of oligonucleotides used for PCR amplification. (Key to oligonucleotides: A_w _= CDC27-Q1W-DIAG2, A_m _= CDC27-Q1M-DIAG2, B = CDC27-B, C = CDC27-SEQ2005, D = CDC27-H, X = CDC1-AB, and Y = CDC1-XY – see Material and methods for sequences). The boxes indicate approximate positions of *cdc27*^+ ^exons. The NotI site shown is found in the *cdc27-Q1 *allele only. Lower part: Genomic DNA prepared from wild-type (WT) and *cdc27-Q1 *(Q1) strains was amplified using the primer pairs shown. The D+B PCR product from *cdc27-Q1 *alone can be digested with NotI (data not shown). The primer pair X and Y amplify an unrelated region of genome, and were included as a control. Molecular weight markers (kb) are shown to the right of the gel. **B. **Wild-type (*cdc27*^+^, left) and *cdc27-Q1 *(right) cells plated on YE medium and incubated for 3 days at 32°C. Mutation of the DPIM did not affect the efficiency of colony formation or growth rate. See text for details.

To confirm this, a fission yeast strain was constructed in which the endogenous *cdc27*^+ ^gene was precisely replaced with the *cdc27-Q1 *mutant allele. In the Cdc27-Q1 mutant protein, the central five amino acids of the DPIM are replaced with alanine, resulting in loss of Cdc27-Pol1 interaction in the two-hybrid system (Figure 4). Construction of the *cdc27-Q1 *strain was achieved by first replacing one copy of *cdc27*^+ ^with *ura4*^+ ^in a diploid strain, before then replacing the *cdc27::ura4*^+ ^allele with *cdc27-Q1 *via 5-FOA counterselection in the *cdc27::ura4*^+ ^haploid carrying *cdc27-Q1 *on a plasmid (see Materials and methods for details). PCR analysis of genomic DNA using primers specific for wild-type *cdc27*^+ ^and *cdc27-Q1 *sequences (see Figure 6 and legend) allowed the identification of haploid strains in which *cdc27-Q1 *was correctly integrated at the endogenous *cdc27*^+ ^locus, and also allowed the *cdc27-Q1 *mutant to be conveniently followed through genetic crosses.

Phenotypic analysis of *cdc27-Q1 *cells, in comparison to an otherwise isogenic *cdc27*^+ ^control, revealed the following: cells carrying the *cdc27-Q1 *allele grew normally at a range of temperatures (18 – 36.5°C), with a generation time indistinguishable from wild-type (110 minutes at 32°C in YE medium). At 32°C, *cdc27-Q1 *cells underwent division at ~ 14.1 μm (compared to wild-type at ~ 14.4 μm). Further analysis showed that the *cdc27-Q1 *cells were indistinguishable from wild-type in all respects examined, including responses to the DNA replication inhibitor hydroxyurea (HU) and the DNA damaging agents methylmethane sulphonate (MMS), camptothecin (CPT), bleomycin sulphate (BMS) and UV light (see Materials and methods for details). The kinetics of cell division arrest in response to HU were also examined, but again no difference was detectable between *cdc27-Q1 *and wild-type strains (data not shown). That cell number increase is arrested in *cdc27-Q1 *cultures following treatment with HU, and that both wild-type and *cdc27-Q1 *cells become highly elongated under these conditions, is indicative of the presence of a functional DNA replication checkpoint in these cells. Taken together, these results strongly suggest that the Pol1-Cdc27 interaction does not play an essential role either during S-phase or in various DNA repair pathways in fission yeast.

The consequences of introducing additional mutations into the *cdc27-Q1 *background were also investigated. *cdc27-Q1 *was crossed to strains carrying mutations in the other three subunits of Pol δ (*pol3-ts3*, *cdc1-P13*, *cdm1Δ*) and in the helicase-endonuclease Dna2 and its associated protein Cdc24 (*dna2-C2 *and *cdc24-M38*). [13,18,30-33]. The double mutant strains were then analysed as described in Materials and methods. In every case examined, the properties of the double mutant with *cdc27-Q1 *were indistinguishable from the single mutant. The *cdc27-Q1 *mutation was also combined with *rad3Δ *[34] and *cds1Δ *[27] alleles, to create *cdc27-Q1 rad3Δ *and *cdc27-Q1 cds1Δ *double mutants, both of which were viable. The Rad3 and Cds1 proteins are key components of various DNA structure checkpoints in fission yeast [35]. That the *cdc27-Q1 *double mutants were viable indicates that *cdc27-Q1 *cells do not require the presence of a functional checkpoint for viability. When wild-type cells are treated with hydroxyurea, activation of Cds1 results in cell cycle arrest and replication fork stabilisation. In the absence of Cds1, however, replication forks are believed to collapse, resulting in loss of viability [35]. The *rad3Δ *and *cds1Δ *double mutants were tested for their sensitivity to HU and CPT, but as before, no differences were observed between single and double mutants with *cdc27-Q1 *(data not shown).

## Discussion

In fission yeast, DNA polymerase δ is a multisubunit complex comprising a large catalytic subunit that is required for chromosomal replication as well as three smaller subunits, two of which are also essential for cell viability [13,18,36]. Understanding how the four subunits of the complex interact with one another, and how they interact with other components of the replication machinery, is an important goal. Once interactions are identified and the sites of interaction precisely mapped, reverse genetic analysis allows determination of the effects of disrupting individual protein-protein interactions on replisome function.

In this paper we show that the C-subunit of fission yeast Pol δ, Cdc27, is able to interact both *in vivo *and *in vitro *with Pol1, the catalytic subunit of Pol α. The Pol1 binding site on Cdc27 has been mapped by *in vivo *and *in vitro *approaches and a region of 40 amino acids, from amino acids 293 – 332, has been shown to be sufficient for binding. Protein sequence alignments of Cdc27 homologues across this 40 amino acid region identify a short protein sequence motif (D -- G --VT) that is highly conserved and which is essential for Pol1 binding. This DNA polymerase interaction motif (DPIM) is flanked by relatively highly charged sequences. Ten basic amino acids are found flanking the DPIM in the fission yeast Cdc27 protein sequence, nine of which are located N-terminal to the central conserved motif. Similarly, there are ten acidic amino acids, all of which lie either within the conserved DPIM or C-terminal to it. Our mutagenesis data clearly implicates several of these charged groups in the binding to Pol1 (Figure 4B). Secondary structure predictions suggest that the conserved DPIM is likely to form a turn or loop, raising the possibility that the sequences on either side of the conserved motif interact with one another. The distribution of positively and negatively charged amino acids in most, though not all, of the Cdc27 homologues in other species (Figure 3) is similar to that in fission yeast. The most obvious exception to this is in the *C. albicans *Cdc27 protein, where six acidic amino acids are found N-terminal, and seven basic amino acids C-terminal, to the DPIM. This organisation of charged residues appears again to be compatible with the notion the sequences on either side of the DPIM may interact with one another.

The Cdc27 protein has an elongated shape, with a frictional ratio of 1.85 [12]. The same is true of its budding yeast orthologue Pol32 which has a frictional ratio of 2.22 [37]. The elongated shape of Cdc27 is due to the C-terminal region of the protein. The N-terminal Cdc1 binding domain, comprising amino acids 1–160 [11], behaves in solution as a globular protein [12]. The protein-protein interaction motif described in this study is the second to be mapped to the extended C-terminal domain. Previously we showed that Cdc27 interacts with PCNA via a conserved sequence at the extreme C-terminus of the protein [11]. Our results indicated that this interaction was essential for cell viability. Consistent with this, Cdc27-PCNA contact is vital for maximal polymerase processivity *in vitro*. However, recently we obtained evidence that the Cdc27-PCNA interaction is a non-essential one (H. Tanaka, G. Ryu, Y.-S. Seo and S.M., submitted).

Recently, the results of a deletion analysis of Pol32, the budding yeast orthologue of Cdc27, were reported [17], including analysis of Pol32-Pol1 interaction. The results of these studies showed that amino acids 270–309 of Pol32 were required for Pol1 binding in the two-hybrid system. This region corresponds to amino acids 286–325 in fission yeast Cdc27 and includes the conserved DPIM sequence (Figure 3). Deletion of amino acids 250–289 (266–305 in Cdc27) greatly reduced Pol1 binding, consistent with the results reported here with the Cdc27-P3, -P4 and -P5 mutants (Figure 4), as did deleting amino acids 310–343 (326–363), consistent with the Cdc27-P7 mutant result. (As expected, the latter Pol32 deletion also disrupted binding to PCNA.) In addition, it was shown that that Pol α-primase and Pol δ could be co-immunoprecipitated following mixing of the purified complexes, but that this interaction was effectively abolished when Pol δ contained the 270–309 deletion of Pol32 rather than the full-length protein [17].

In this paper, cells expressing the DPIM mutant protein Cdc27-Q1 were shown to be no more sensitive then wild-type to HU, MMS, CPT, UV and BMS (see Results), while analysis of budding yeast cells expressing Pol32 lacking the DPIM (270–309 deletion) showed them to be no more sensitive than wild-type to HU and UV, and to show normal rates of mutagenesis following UV exposure [17]. Indeed, in all situations examined, *S. pombe cdc27-Q1 *cells were indistinguishable from wild-type. No genetic interactions were observed between *cdc27-Q1 *and various other DNA replication or checkpoint mutants, including the key checkpoint kinase Rad3.

In conclusion, while the interaction between Pol α-primase and Pol δ mediated via Cdc27 could play an important role in coordinating the events of lagging strand synthesis, we have yet to obtain any evidence that this is the case. This raises two possibilities. First, that the observed interaction does not play an important role in chromosomal replication. We believe that this is unlikely to be the case, given the high degree of conservation of the DPIM sequence across evolution, in a region of the Cdc27 protein that is very poorly conserved at the primary sequence level. The second possibility is that there are multiple redundant interactions within the lagging strand machinery. In this case, an important role for the Cdc27-Pol1 interaction might only be uncovered when another protein-protein interaction is perturbed, in which case, the *cdc27-Q1 *mutation might be expected to be synthetically lethal or sick with a mutant that disrupted the overlapping redundant function. With the genetic tools available in yeast, this is a hypothesis that is readily testable.

## Conclusions

In fission yeast, interaction between Pol α and Pol δ is mediated, at least in part, by direct binding of the Pol δ C-subunit Cdc27 to the Pol α catalytic subunit Pol1, and requires the presence of a short sequence motif (DPIM) in the C-terminal region of Cdc27. The DPIM is conserved in all known Cdc27 orthologues. Despite this, it has not been possible to identify any phenotypic consequences associated with deletion of the DPIM sequence, raising the possibility that the observed interaction does not play a crucial role *in vivo*.

## Methods

### Yeast strains, media and methods

All fission yeast strains were as described previously, except for *pol1-H4 *[27] and *pol1-1 *[26], which were obtained from Dr J. Hayles (CR-UK, London, U.K.), and *pol1-ts13 *[25], which was obtained from Dr T.S.F. Wang (Stanford, U.S.A.). Note that this allele was originally designated *polα-ts13 *but is correctly renamed here to bring it in line with accepted nomenclature standards (see  for further details). *S. pombe *media and techniques were essentially as described [38], with the following exceptions. Routine transformation of *S. pombe *was carried out by electroporation [39], whereas transformation for PCR-based gene targeting was accomplished using a modified lithium acetate method [40]. For two-hybrid analysis. *S. cerevisiae *CTY10-5d (*MATa ade2 met- trp1-901 leu2-3-112 his3-Δ200 gal4-gal80-URA3::lexA-LacZ*) was used [18]. *S. cerevisiae *was cultured in YPDA and SD medium.

### Plasmids for two-hybrid assay

Two-hybrid interactions were monitored using the Gal4 transcription activation domain (prey) plasmid pACT2 (Clontech), the LexA DNA binding domain (bait) plasmid pBTM116, and *S. cerevisiae lexA op-lacZ *strain CTY10-5d, as described previously [18].

The Pol1 bait plasmid pBTM116-Pol1-(278–527) was constructed by amplifying sequences encoding amino acids 278–527 from plasmid pTZ19R-Pol1 (prepared by subcloning a 5925 bp SmaI-PstI fragment encompassing the entire *pol1*^+ ^gene from *S. pombe *cosmid SPAC3H5, see , into plasmid pTZ19R) using oligonucleotides POL15 (5'-GTGTGGTTTGGGATCCCCCTATCACCAATGACACCTTTA-3') and POL13-2 (5'-GTGTGGTTTGGGATCCTACATCACCGTCATTGGAGGCGT-3'), restricting the PCR product with BamHI (sites underlined), cloning to pTZ19R (Fermentas) and sequencing to confirm the absence of errors. The 763 bp BamHI fragment was then transferred to pBTM116, to generate pBTM116-Pol1(278–527). Plasmid pBTM116-Pol1-TS13(278–527) was produced in a similar manner, except that the starting PCR template was genomic DNA prepared from *pol1-ts13 *cells [25], the final product thereby containing a 9 bp deletion in the *pol1*^+ ^ORF, resulting in the loss of coding capacity for amino acids L470, S471 and R472. Plasmids expressing C-terminally truncated Pol1(278–527) proteins were constructed by PCR amplification using oligo POL15 in conjunction with POL25-3 (5'-GTGTGGTTTGGGATCCTAAGGACCCATAACTCTTCTACT-3'), POL13-487 (5'-GTGTGGTTTGGGATCCTAAAAATTTGGTTGTTGTATTTT-3'), POL1-497 (5'-GTGTGGTTTGGGATCCTACCGGCACCAACTAGCATTTTT-3') or POL1-507 (5'-GTGTGGTTTGGGATCCTAGTTCTGAGGTGACGAACATCC-3'), cloning directly to pBTM116 following BamHI cleavage of the PCR product, and sequencing. The resulting constructs encoded the following proteins as Lex A fusions: Pol1(278–477), Pol1(278–487), Pol1(278–497) and Pol1(278–507). A construct expressing an N-terminally truncated Pol1(278-527) protein, designated Pol1(328–527), was generated by amplification using POL15-2 (5'-GTGTGGTTTGGGATCCCCGGCTCATTGTGTCTATTTGGC-3') with POL13-2 (above). Amplification with POL15-2 and POL25-3 generated a construct with the potential to express a LexA-Pol1 protein truncated at both ends: LexA-Pol1(328–477).

Sequences encoding the C-terminal region from human p66/KIAA0039 were amplified by PCR from plasmid pET19b-p66 (a gift of Dr P. Hughes, Villejuif, France) using either oligo P66-51 (5'-GTGTGGTTTGGGATCCCCTCAGAACAAGCAGTGAAAGAA-3') or P66-52 (5'-GTGTGGTTTGGGATCCCGTCTCCACCTCTTGAACCAGTG-3') with P66-3 (5'-GTGTGGTTTGGGATCCTTGGTCTTCACCCTTGACCACTC-3'). The PCR products were then restricted with BamHI, cloned to pACT2 and sequenced. The resulting plasmids encode, as Gal4 AD fusions, either the entire C-terminal domain of p66/KIAA0039 (amino acids 253–466, oligos P66-51 and P66-3) or a shorter region (amino acids 356–466, oligos P66-51 and P66-3). Sequences encoding the interacting domain of the catalytic domain of human Pol α were amplified from plasmid pBR322-Pol α (a gift of Dr T. Wang, Stanford, USA) using oligos HPOL1-5 (5'-GTGTGGTTTGGGATCCCCAAAGGGACCGTGTCCTACTTA-3') and HPOL1-3 (5'-GTGTGGTTTGGGATCCTACATCACGACAAGCGGTGGTGG-3'), restricted with BamHI, cloned to pBTM116 and sequenced. The resulting plasmid encodes amino acids 291–540 of the human protein as a LexA fusion.

The Cdc27 prey plasmids were constructed in pACT2 (Clontech) and have been previously described, with the exception of mutants Cdc27-273-352-P1 through -P7, and Cdc27-273-352-Q1. The first four of these (P1-P4) were generated by PCR amplification from the pREP3X-Cdc27-P1 to -Cdc27-P4 plasmids described below. Oligonucleotides for PCR, with BamHI sites underlined: PMUT5 (5'-GTTTGTTGGTGGATCCCCACCGAAGCAAAATCTGCTGCA-3') and PMUT3 (5'-GTTGTGGGTGGGATCCTACTTCTTAGTTGCAATGTTTAC-3'). Mutant Q1 was generated by PCR amplification with same primers but using pHBLA-Cdc27-Q1 (below) as template. Plasmids expressing mutants P5-P7 were generated by overlap extension PCR using PMUT5 and PMUT3 together with the following mutagenic oligonucleotides (shown with mutated sequence underlined in top strand oligo): CDC27-P5F (5'-AAAGAAAAGTTGCAGCGTACGCGACAACGAAAG-3'), CDC27-P5R (5'-CTTTCGTTGTCGCGTACGCTGCAACTTTTCTTT-3'), CDC27-P6F (5'-TTGGTTACTAAGGCAGCAGCAGTCTGGGAATCA-3'), CDC27-P6R (5'-TGATTCCCAGACTGCTGCTGCCTTAGTAACCAA-3'), CDC27-P7F (5'-GAATCATTTTCTGCAGCTGCAAACATCTCAACT-3'), CDC27-P7R (5'-AGTTGAGATGTTTGCAGCTGCAGAAAATGATTC-3').

### Two-hybrid assays

Quantitative data for β-galactosidase activity was obtained as described previously [18] and is expressed in Miller units [41]. To analyse prey protein levels, yeast total protein extracts, prepared from the same cultures used for β-galactosidase assay, were subjected to SDS-PAGE and immunoblotted using antibodies against the HA epitope (12CA5, Roche Applied Science) present in proteins expressed from pACT2.

### Expression and purification of recombinant H6-Pol1 (278–527)

The Pol1 (278–527) domain was expressed in *E. coli *with an N-terminal MRGSH_6_-tag to facilitate purification and detection. To achieve this, the 763 bp Pol1 (278–527)-encoding BamHI fragment described above was cloned into pQE32 (Qiagen). The resulting plasmid, pQE32-Pol1 (278–527), was transformed into *E. coli *M15 (pREP4) and recombinant protein expression induced in 500 ml cultures (OD_600 nm _of 0.6) by addition of IPTG to a final concentration of 1 mM. Following incubation for 4 hours at 37°C, the cells were pelleted and the pellet resuspended in 40 ml of ice-cold buffer A (150 mM NaCl, 50 mM Tris-HCl pH 8.0, 20 mM imidazole) containing EDTA-free Complete™ inhibitors (Roche Applied Science) and 1 mM PMSF. The cells were lysed by sonication, then centrifuged at 25000 g for 15 minute at 4°C. The soluble supernatant was added to 4 ml of 50% (v/v) Ni-NTA agarose (Qiagen) in buffer A, mixed on a wheel at 4°C for one hour, then packed into a disposable chromatography column at 4°C. The column was drained of the flow-through and subsequently washed with 100 ml of buffer B (1 M NaCl, 50 mM Tris-HCl pH 8.0, 20 mM imidazole) containing Complete™ inhibitors and PMSF, then 100 ml of buffer A containing Complete™ inhibitors and PMSF, before the bound H6-Pol1 protein was eluted using 4 ml of buffer C (250 mM imidazole, 50 mM Tris-HCl pH 8.0). Following elution, samples were analysed by SDS-PAGE and the peak fractions pooled and dialysed overnight in PBS at 4°C. Protein concentration was determined by BCA assay (Pierce) versus BSA standards.

### Binding assays

GST and GST-Cdc27-273-352 fusion proteins were prepared as described previously [11] from plasmid pGEX6P-1B, a modified version of pGEX6P-1 (Amersham Pharmacia) in which the reading frame of the polylinker is altered (S.M., unpublished). Sequences encoding wild-type and mutant forms of Cdc27-273-352 were subcloned into this vector from the equivalent pACT2 two-hybrid constructs described above. Binding assays were performed by mixing ~ 30 pmol of GST or GST-Cdc27-273-352 with 90 pmol of H6-Pol1 in PBS containing 1.0% Triton X100 for 1 hour at 4°C on a rotating wheel. The GST proteins were then precipitated using Glutathione Sepharose™ 4 Fast Flow resin (Amersham Biosciences) for 30 minutes at 4°C on a rotating wheel. Following extensive washing with PBS containing 0.1 – 1.0% Triton X100, SDS-PAGE sample buffer was added and the samples heated at 95°C for 5 minutes. Bound H6-Pol1 was visualised following electrophoresis of 15% SDS-PAGE gels by PAGE Blue G90 (Fluka) staining or by immunoblotting with anti-MRGS antibodies (Qiagen).

### Construction of Pol1-13Myc strain

PCR-based gene targeting was used to tag the chromosomal *pol1*^+ ^locus in an otherwise wild-type *leu1-32 ura4-D18 *h^- ^strain with sequences encoding thirteen copies of the 9E10 (c-myc) mAb epitope, such that the encoded Pol1 protein (termed Pol1-13Myc) carries the 9E10 epitopes at its C-terminus. Oligonucleotides for amplification from pFA6a-13Myc-kanMX6 [40] were as follows: POL1-13MYC-5 (5'-TGCCATCAACAAAAATATCTCTCGAATAATGAACAAAAATGCGCGTGAATTTGTAGATATGGGACTGATATTTTCATCGCGGATCCCCGGGTTAATTAA-3', with plasmid-specific sequence underlined) and POL1-13MYC-3 (5'-GGCAATTCCCAAGTCTTTGAAACAGGTATTCCCATCAACATTTCTTGTACTGCATGAGCAAATATCTGTTCGAGGTGTCGAATTCGAGCTCGTTTAAAC-3'). Following transformation of ~ 10 μg of PCR product, correct G418-resistant integrants were identified by PCR amplification of genomic DNA using primer FCGPOL1-5 (5'-ATGTCGTGGAAGCGTTCATT-3') within the *pol1*^+ ^ORF and KAN269R (5'-GATCGCAGTGGTGAGTAACCATGCATCATC-3') within the *kanMX6 *cassette, and by Western blotting using the mouse anti-Myc mAb 9E10 (Roche).

### Peptide binding assays

Peptides were synthesized by Mimotopes (Australia). All peptides were synthesized to contain the sequence biotin-SGSG at the N-terminus. Peptide sequences: SpA AAPDEPQEIIKSVSGGKRRG; SpB PQEIIKSVSGGKRRGKRKVK; SpC KSVSGGKRRGKRKVKKYATT; SpD GKRRGKRKVKKYATTKDEEG: SpE KRKVKKYATTKDEEGFLVTK; HsA PKTEPEPPSVKSSSGENKRK; HsB EPPSVKSSSGENKRKRKRVL; HsC KSSSGENKRKRKRVLKSKTY; HsD ENKRKRKRVLKSKTYLDGEG; HsE RKRVLKSKTYLDGEGCIVTE. For binding assays, the peptides were initially bound to 10 μl Streptavidin-agarose beads in PBS in a final volume of 100 μl at room temperature for 1 hour with gentle agitation. After binding, the beads were washed 3 times with 100 μl PBS. Protein extracts (100 μg of fission yeast extract) were added to the beads, and incubated at 4°C for 1 hr with gentle agitation. The beads were pelleted by centrifugation at 2000 rpm for 3 minutes, and subsequently washed extensively with NP40 buffer (50 mM Tris-HCl pH 8.0, 150 mM NaCl, 1% (v/v) NP-40), prior to final resuspension with 20 μl of SDS loading buffer. Following SDS-PAGE, Pol1-13Myc was visualised by immunoblotting with the mouse anti-Myc mAb 9E10 (Roche). Fission yeast protein extracts were prepared from 8 × 10^7 ^cells in mid-exponential growth in EMM medium. Cells were harvested, washed once in STOP buffer (150 mM NaCl, 50 mM NaF, 10 mM EDTA, 1 mM NaN_3_) and disrupted in buffer A (10 mM sodium phosphate buffer pH 7.0, 1% Triton, 0.1% SDS, 1 mM EDTA, 150 mM NaCl, 1 mM PMSF) supplemented with Complete™ protease inhibitors (Roche), using a bead beater (Hybaid Ribolyser). Protein concentrations were determined at 595 nm using the BioRad protein assay reagent according to the manufacturer's instructions, with BSA as standard.

### Plasmids expressing DPIM mutants

Plasmids pREP3X-Cdc27-P1 to -Cdc27-P4 were constructed by cloning mutagenised *cdc27*^+ ^cDNAs (SalI-BamHI) from pTZ19R-Cdc27-cDNA [18] into pREP3X. Mutagenesis of pTZ19R-Cdc27-cDNA was accomplished using the MutaGene II mutagenesis kit (BioRad) according to the manufacturer's instructions. Oligonucleotides with mutated residues underlined: CDC27-P1 (5'-AAAAGTACGCGACAACGAAAGCCGCAGCAGGATTCTTGGTTACTAAGG-3'), CDC27-P2 (5'-CGACAACGAAAGATGAAGAAGCCGCCTTGGTTACTAAGGAAGAAG-3'), CDC27-P3 (5'-TCAAATCCGTATCCGGTGGAGCCGCAGCAGGGAAAAGAAAAGTTAAAAAG-3'), CDC27-P4 (5'-CCGGTGGAAAGAGACGTGGGGCCGCAGCAGTTAAAAAGTACGCGACAAC-3'). The resulting mutant alleles were tested for function by transforming a *cdc27*^+^*/cdc27::his7*^+^*leu1-32/leu1-32 ura4-D18/ura4-D18 his7-366/his7-366 ade6-M210/ade6-M216 h*^-^/*h*^+ ^diploid [11], transferring the transformants onto malt extract medium to induce sporulation, before finally plating the helicase-treated spores onto EMM plates supplemented with 5 μg/ml thiamine with/without histidine, at 32°C.

### Construction of DPIM mutant strain

A 3.1 kb HindIII-BamHI genomic DNA fragment carrying the *cdc27*^+ ^gene was first cloned into pTZ19R to make pHB-Cdc27. This vector was then modified by addition of the *S. cerevisiae LEU2 *gene and *S. pombe ars1*, to make pHBLA-Cdc27. The *cdc27*^+ ^gene was then subjected to oligonucleotide-directed *in vitro *mutagenesis using the QuikChange method (Stratagene) with oligonucleotides CDC27-QC1 (5'-GTACGCGACAACGAAAGCTGCAgcggccgcCTTGGTTACTAAGGAAGAAG-3', with mutated sequence underlined and NotI site in lower case) and CDC27-QC2 (5'-CTTCTTCCTTAGTAACCAAGgcggccgcTGCAGCTTTCGTTGTCGCGTAC-3'), to create plasmid pHBLA-Cdc27-Q1. This was then was transformed into a *cdc27*^+^/*cdc27::ura4*^+ ^diploid, and *cdc27::ura4*^+ ^(pHBLA-Cdc27-Q1) haploids obtained following sporulation and regrowth. These were then plated on YE plates containing 1 mg/ml 5-FOA. 5-FOA resistant colonies were identified, purified and characterised by PCR amplification of genomic DNA (prepared using the method of Bähler and coworkers [40]), to ensure loss of the chromosomal *ura4*^+ ^marker, its replacement with *cdc27-Q1*, and loss of the pHBLA-Cdc27-Q1. Oligonucleotides for diagnostic PCR shown in Figure 6: CDC27-Q1W-DIAG2 (5'-GCGACAACGAAAGATGAAGAAGGATTC-3'; note that the underlined region anneals only to the wild-type *cdc27*^+ ^and not *cdc27-Q1*); CDC27-Q1M-DIAG2 (5'-GCGACAACGAAAGCTGCAGgcggccgcC-3'; underlined region anneals only to *cdc27-Q1 *and not to *cdc27*^+^; NotI site in lower case); CDC27-H (5'-ACTGGTAGAATTGCGTTCGCGCTC-3'); CDC27-B (5'-TCTAGGATCAGAGTGAACTGATTG-3'); CDC27-SEQ2005 (5'-AGGTTGTACTAACATTAACAG-3'). The resulting strain, *cdc27-Q1 leu1-32 ura4-D18 his7-366 ade6-M216 *h^- ^was then analysed alongside the wild-type *leu1-32 ura4-D18 his7-366 ade6-M216 *h^-^, as described below.

### Phenotypic analysis

Cells were grown to mid-exponential phase (~ 5 × 10^6 ^cells/ml) in YE medium and ~ 2000 cells plated on YE medium supplemented with varying concentrations of hydroxyurea (2.5, 5, 7.5, 10, 12.5 mM), methylmethanesulphonate (0.01, 0.005, 0.0025, 0.001, 0.0005, 0.00025, 0.0001%), bleomycin sulphate (2.5, 5, 7.5, 10, 12.5 mU/ml), or camptothecin (4, 4.5, 5, 5, 5.5, 6, 6.5, 7, 7.5, 8 μM). Cells were also plated on plates containing sub-lethal doses of both HU and CPT, specifically 7.5 mM HU with either 5.5 or 6 μM CPT, or 10 mM HU with either 5.5 or 6 μM CPT. To analyse the effects of HU treatment in liquid culture, HU was added to EMM medium to a final concentration of 12 mM. Cell number per ml of culture was monitored using a Coulter Z1 electronic particle counterTo test sensitivity to UV, 1000 cells were plated on EMM plates, allowed to dry for 20 minutes, then irradiated using either a Stratalinker UV source (Stratagene) over the range 0 – 250 J/m^2^. Following UV treatment, plates were placed immediately in the dark to avoid photoreversal. For all treatments, the efficiency of colony formation was determined after 4 days growth at 32°C. Growth rate (YE medium, 32°C) was determined by cell counting using a particle counter. Cell length at cell division was determined using a graduated eyepiece.

### DPIM double mutants

Double mutants were constructed by standard methods, with the *cdc27-Q1 *allele being identified by PCR analysis of genomic DNA using CDC27-Q1W-DIAG2 and CDC27-Q1M-DIAG2 oligonucleotides described above. Double mutants were created with the following: *pol3-ts3 *[30], *cdc1-P13 *[42], *cdm1::ura4*^+^[13], *cdc24-M38 *[43], *dna2-C2 *[31], *rad3::ura4*^+ ^[34] and *cds1::ura4*^+ ^[27].

## List of abbreviations used

DPIM (DNA polymerase interaction motif); PCNA (proliferating cell nuclear antigen).

## Authors' contributions

In Edinburgh, SM conceived of the study, performed some of the experimental work and prepared and revised the final manuscript, while FG performed the remainder of the experimental work. In Dundee, EW and JRGP designed and carried out the peptide binding studies. All four authors read and approved the manuscript.
